# ﻿Five new species of *Synagelides* Strand, 1906 from China (Araneae, Salticidae)

**DOI:** 10.3897/zookeys.1102.76800

**Published:** 2022-05-19

**Authors:** Ke-ke Liu, Zi-Yi Zhao, Yong-hong Xiao, Xian-Jin Peng

**Affiliations:** 1 College of Life Science, Jinggangshan University, Ji’an 343009, Jiangxi, China; 2 Key Laboratory of Agricultural Environmental Pollution Prevention and Control in Red Soil Hilly Region of Jiangxi Province, Jinggangshan University, Ji’an, 343009, Jiangxi, China; 3 College of Life Science, Hunan Normal University, Changsha 410081, Hunan, China

**Keywords:** Ant-like, Gansu Province, Jiangxi Province, Jumping spider, taxonomy

## Abstract

Five new species of salticids were collected from China: *Synagelidesemangou* Liu, **sp. nov.** (♂, ♀) from Gansu province, and *S.jinding* Liu, **sp. nov.** (♂), *S.serratus* Liu, **sp. nov.** (♂, ♀), *S.shuqiang* Liu, **sp. nov.** (♂), and *S.triangulatus* Liu, **sp. nov.** (♀) from Jiangxi Province. All species are described and illustrated with photographs and SEM micrographs, and their distributions are also mapped.

## ﻿Introduction

The family Salticidae, or jumping spiders, is the most diverse spider family worldwide. It consists of 6368 species in 659 genera (WSC 2021). Of these, 572 species in 123 genera have been reported from China. Of these 123 genera, *Phintella* Strand, 1906 (30 species) and *Synagelides* Strand, 1906 (30 species) have been reported as being the most diverse in China (Li and Lin 2021; WSC 2021). The spider species of the genus *Synagelides* Strand, 1906 are characterized by their ant-like bodies and conspicuously thickened femur I. They are usually found in a wide range of habitats such as grasslands and forests and living in bark, brush, leaf litter, humus, leaves, forest canopies, and under rocks. To date, there are 30 species (more than half of the total number of species) known from China, and eight species are known by only one sex (WSC 2021). Most of these *Synagelides* species are described from the southern provinces, and only two species were recorded from the northern provinces.

When examining spider specimens collected from Qinghai, Gansu, Hebei, Shanxi, and Jiangxi provinces, five new *Synagelides* species were identified, and they are described here: *Synagelidesemangou* sp. nov., *S.jinding* sp. nov., *S.serratus* sp. nov., *S.shuqiang* sp. nov., and *S.triangulatus* sp. nov.

## ﻿Materials and methods

Specimens were examined using a Zeiss Stereo Discovery V12 stereomicroscope with a Zeiss Axio Cam HRc. Both the male palps and female copulatory organs were dissected and examined in 80–85% ethanol. The vulvae were cleaned in pancreatin. All the specimens were photographed with an Olympus CX43 compound microscope with a KUY NICE CCD (Beijing Tiannuoxiang Scientific Instrument Co., Ltd, China). For SEM photographs, the specimens were dried under natural conditions, sprayed with gold with a small ion-sputtering apparatus ETD-2000 (Beijing Yilibotong Technology Development Co., Ltd, China), or used without coating, and photographed with a Zeiss EVO LS15 (Carl Zeiss AG, Germany) scanning electron microscope. Images were edited using the ImagineView software package and the Smart SEM User Interface.

All measurements were made by using a stereomicroscope with AxioVision SE64 Rel. 4.8.3 software and are given in millimeters. Leg measurements are given as the total length (femur, patella, tibia, metatarsus, tarsus). Specimens were put in separate bottles with a collection number and a serial number, such as 20200504-1, sp6. Holotype and paratype are labeled by red and yellow cards, respectively. All specimens are deposited in the Animal Specimen Museum, College of Life Science, Jinggangshan University (**ASM-JGSU**).

Terminology of male and female copulatory organs follows Liu et al. (2017), Kanesharatnam and Benjamin (2020), and Wang et al. (2020). The abbreviations used in the text and figures are:

**Eyes**

**ALE** anterior lateral eye;

**AME** anterior median eye;

**PLE** posterior lateral eye;

**PME** posterior median eye;

**MOA** median ocular area.

**Legs**

**ti** tibia;

**pv** proventral;

**rv** retroventral;

**met** metatarsus.

**Male palp**

**DTA** dorso-prolateral tibial apophysis;

**Em** embolus;

**PCA** postero-prolateral cymbial apophysis;

**RCA** postero-retrolateral cymbial apophysis;

**RTA** retrolateral tibial apophysis;

**SD** sperm duct;

**SS** scale-like serrations;

**TA** terminal apophysis;

**VFA** ventral femoral apophysis.

**Epigyne**

**AR** atrial rim;

**At** atrium;

**CD** copulatory duct;

**CO** copulatory opening;

**EH** epigynal hood;

**FD** fertilization duct;

**GA** glandular appendages;

**MS** median septum;

**Spe** spermatheca.

## ﻿Taxonomy

### ﻿Family Salticidae Blackwall, 1841

**Tribe Agoriini Simon, 1901 (*sensu*Maddison 2015)**

#### Genus *Synagelides* Strand, 1906

##### ﻿ Synagelides emangou

Taxon classificationAnimaliaAraneaeSalticidae

Liu sp. nov.

556D4682-E121-5682-879F-1D76D9539129

http://zoobank.org/CCBBBC83-FE2D-4FCD-9EF8-52CF934B8B17

Figs 1 , 2 , 3

###### Material examined.

***Holotype*** ♂, 33°57'23.50"N, 104°25'25.56"E, 1795 m, near parking lot, Emangou Scenic Area, Lugangtou Village, Xinchengzi Town, Tanchang County, Longnan City, Gansu Province, China, 28 July 2021, K. Liu, Y. Ying & C. Xu. ***Paratype*** 2 ♂, 1 ♀, the same data as holotype.

###### Etymology.

The name is taken from the type locality, Emangou Scenic Area; noun in apposition.

###### Diagnosis.

**Figure 1.**
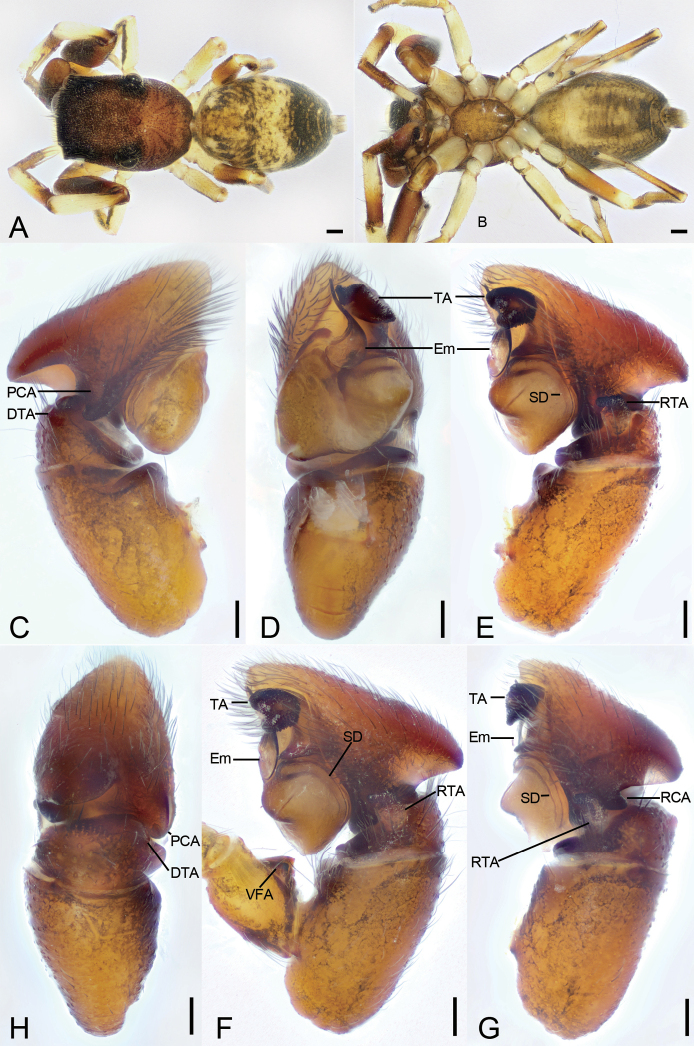
*Synagelidesemangou* sp. nov., holotype male **A** habitus, dorsal view **B** same, ventral view **C** palp, prolateral view **D** same, ventral view **E** same, retrolateral view **F** same, detail of ventral femoral apophysis, retrolateral view **G** same, retrolateral view, slightly dorsal **H** same, dorsal view. Abbreviations: DTA – dorso-prolateral tibial apophysis, Em – embolus, PCA – postero-prolateral cymbial apophysis, RCA – postero-retrolateral cymbial apophysis, RTA – retrolateral tibial apophysis, SD – sperm duct, TA – terminal apophysis, VFA – ventral femoral apophysis. Scale bars: 0.2 mm (**A, B**); 0.05 mm (**C–E, G, H**); 0.1 mm (**F**).

**Figure 2.**
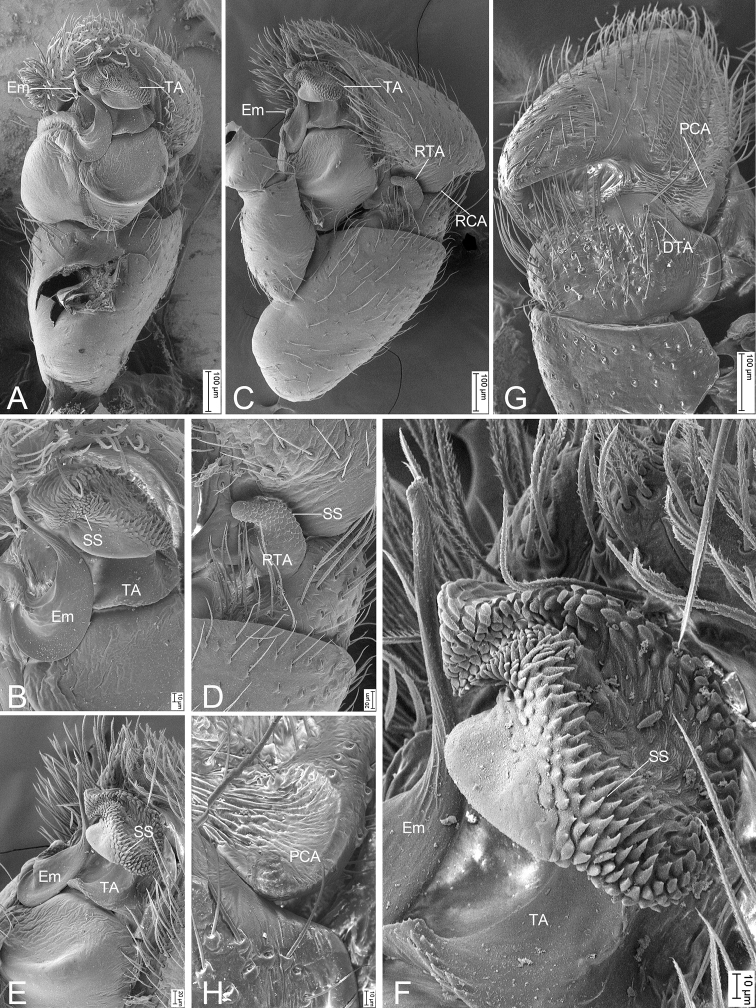
*Synagelidesemangou* sp. nov., SEMs of male paratype **A** palp, ventral view, slightly frontal **B** same, detail of terminal apophysis and embolus, ventral view, slightly frontal **C** same, retrolateral view **D** same, detail of retrolateral tibial apophysis, retrolateral view **E** same, detail of terminal apophysis and embolus, retrolateral view **F** same, detail of terminal apophysis and embolus, retrolateral view **G** same, dorsal view **H** same, detail of postero-prolateral cymbial apophysis, dorsal view. Abbreviations: DTA – dorso-prolateral tibial apophysis, Em – embolus, PCA – postero-prolateral cymbial apophysis, RCA – postero-retrolateral cymbial apophysis, RTA – retrolateral tibial apophysis, SS – scale-like serrations, TA – terminal apophysis.

**Figure 3.**
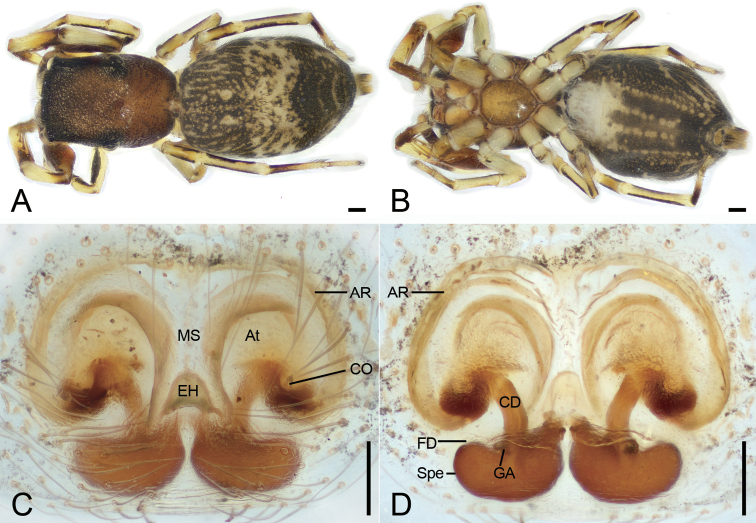
*Synagelidesemangou* sp. nov., female paratype **A** habitus, dorsal view **B** same, ventral view **C** epigyne, ventral view **D** same, dorsal view. Abbreviations: AR – atrial rim, At – atrium, CD – copulatory duct, CO – copulatory opening, EH – epigynal hood, FD – fertilization duct, GA – glandular appendages, MS – median septum, Spe – spermatheca. Scale bars: 0.2 mm (**A, B**); 0.05 mm (**C, D**).

The males of this species are similar to males of *Synagelideszhaoi* Peng, Li & Chen, 2003 (see Peng et al. 2003: 249, figs 2–5) in having a thick ventral femoral apophysis in retrolateral view and a golf-club-shaped embolus in ventral view, but differs from it in having (Figs 1C–H, 2) a triangular tibia (vs saddle-shaped), a forcipate retrolateral tibial apophysis (vs short and horn-shaped), the broad postero-prolateral cymbial apophysis (vs relatively narrow), a C-shaped terminal apophysis (vs S-shaped), and the mastoid tegular in retrolateral view (vs S-shaped). The female resembles *S.zhaoi* (see Peng et al. 2003: 249, figs 6, 7) in having a nose-shaped median septum and the C-shaped atrial rims, but it can be easily recognized by (Fig. 3C, D) the relatively broad, tube-shaped copulatory ducts (vs slender) and the swollen spermathecae (vs relatively thin).

###### Description.

**Male** (holotype, sp7-20210728-1, red label). ***Habitus*** as in Fig. 1A, B. Total length 3.59. Carapace 1.70 long, 1.23 wide. Eye sizes and interdistances: AME 0.31; ALE 0.15; PME 0.09; PLE 0.16; AME–AME 0.08; AME–ALE 0.08; PME–PME 0.90; ALE–ALE 1.09; PME–PLE 0.36; PLE–PLE 1.00; ALE–PLE 0.80; AME–PME 0.46; AME–PLE 0.91. MOA: 0.66 long; 0.66 anterior width, 1.02 posterior width. Fovea (Fig. 1A) round, hollowed. Chelicerae (Fig. 1B) with two promarginal teeth (proximal larger) and one large laminar retromarginal teeth. Sternum (Fig. 1B) shield-shaped, longer than wide, posterior end arch-shaped, smooth. Leg measurements: I 4.2 (1.29, 1.03, 1.05, 0.45, 0.38); II 2.62 (0.85, 0.41, 0.54, 0.55, 0.27); III 2.52 (0.74, 0.44, 0.58, 0.52, 0.24); IV 3.89 (1.13, 0.53, 0.95, 0.9, 0.38). Femur width: I 0.50; II 0.26; III 0.27; IV 0.35. Leg spination (Fig. 1A, B): I tipv 1-2-1, rv 1-2-1; Met pv 0-1-1, rv 0-1-1. Pedicel 0.11. Abdomen 1.80 long, 1.25 wide.

***Coloration*** (Fig. 1A, B). Carapace reddish brown, anterior part darker than posterior, posteriorly with radial grooves and 14–16 rows of short, white setae. Endites yellow, mottled. Labium dark yellow-brown, anteriorly with a single row of strong setae. Sternum, yellow-brown, mottled, with dark brown mottled stripes around margin. Legs: trochanter I yellow-brown, trochanters II–IV yellow, with dark brown stripe; femur I dark yellow-brown, femora II–IV yellow, with distinct prolateral and retrolateral dark brown stripes; patellae, tibiae, and metatarsi yellow, with dark brown lateral stripes; tarsi yellowish, proximal part darker than distal. Abdomen yellow to dark brown, anterior part yellow, mottled, posterior part dark brown with four paler chevron-shaped stripes medially; venter with a U-shaped dark yellow-brown marking postero-medially. Spinnerets yellowish brown, mottled.

***Palp*** (Figs 1C–H, 2). Femur with a thick, strong tooth-like ventral apophysis. Patella swollen, with a ratio of ca 1.85 between its length and width. Tibia small and narrow with a forcipate stubby retrolateral apophysis, less than 1/2 length of cymbium, with numerous scale-like serrations on apical surface. Cymbium bullet-shaped in dorsal view, with a strong sclerotized postero-retrolateral and a long strong postero-prolateral apophysis. Tegulum broad, C-shaped in ventral view, with a clear mastoid apophysis in retrolateral view. Terminal apophysis arising from antero-retrolateral part of tegulum, strongly sclerotized, C-shaped in retrolateral view, with abundant little scale-like serrations on surface. Embolus golf-club-shaped in ventral view, longer than terminal apophysis, with very broad basal part and whip-shaped apical part.

**Female** (paratype, sp7-20210728-1, yellow label). ***Habitus*** as in Fig. 3A, B. As in male, except as noted. Total length 3.79. Carapace 1.51 long, 1.16 wide. Eye sizes and interdistances: AME 0.31; ALE 0.18; PME 0.06; PLE 0.21; AME–AME 0.05; AME–ALE 0.08; PME–PME 0.83; ALE–ALE 1.02; PME–PLE 0.32; PLE–PLE 1.00; ALE–PLE 0.67; AME–PME 0.42; AME–PLE 0.66. MOA: 0.66 long; 0.67 anterior width, 0.93 posterior width. Chelicerae (Fig. 3B) with two promarginal teeth (slightly separated, proximal larger) and one large triangular retromarginal teeth. Sternum (Fig. 3B), posterior end triangular, relatively blunt. Leg measurements: I 3.17 (0.91, 0.76, 0.82, 0.35, 0.33); II2.3 (0.66, 0.34, 0.59, 0.37, 0.34); III 2.4 (0.71, 0.34, 0.52, 0.53, 0.3); IV 3.53 (1, 0.51, 0.89, 0.73, 0.4). Femur width: I 0.31; II 0.20; III 0.21; IV 0.21. Pedicel 0.11. Leg spination (Fig. 3A, B): I tipv 2-2-0, rv 2-2-0; Met pv 1-0-1, rv 1-0-1. Pedicel 0.21. Abdomen 2.11 long, 1.44 wide.

***Coloration*** (Fig. 3A, B). Darker than male. Ventral abdomen with three broad longitudinal dark brown stripes, posteriorly fusing.

***Epigyne*** (Fig. 3C, D). Epigynal plate mask-shaped, with a nose-shaped median septum. Epigynal hood bell-shaped, arising from posterior part of median septum. Atrium relatively large, nearly covering 1/3 of epigynal field. Atrial rims C-shaped, slightly sclerotized, located at bilateral parts of epigyne. Copulatory ducts short, tube-shaped, with a slight curve posteriorly, connecting with submedial part of spermathecae. Glandular appendages very short, near the posterior copulatory ducts. Spermathecae kidney-shaped, swollen, slightly separated. Fertilization ducts relatively long, >2/3 length of spermathecae, transversely extended.

###### Distribution.

Known only from the type locality in Gansu Province, China (Fig. 13).

##### ﻿ Synagelides jinding

Taxon classificationAnimaliaAraneaeSalticidae

Liu sp. nov.

3721D039-DED3-579F-B84A-005137D9E155

http://zoobank.org/5C75C451-4BB0-4602-A8CF-7DC9DC6A479C

Figs 4 , 5

###### Material examined.

***Holotype*** ♂, 27°26'45.19"N, 114°11'17.53"E, 1223 m, Tupingao area, near Ropeway, Wugong Mountain National Forest Park, Taishan Town, Anfu County, Ji’an City, Jiangxi Province, China, 4 May 2021, K. Liu, Y. Ying, C. Xu & Q. Xiao leg.

###### Etymology.

The name is taken from the famous Jinding Scenic Spot, which is very close to Tupingao area in the Wugong Mountain National Forest Park; noun in apposition.

###### Diagnosis.

**Figure 4.**
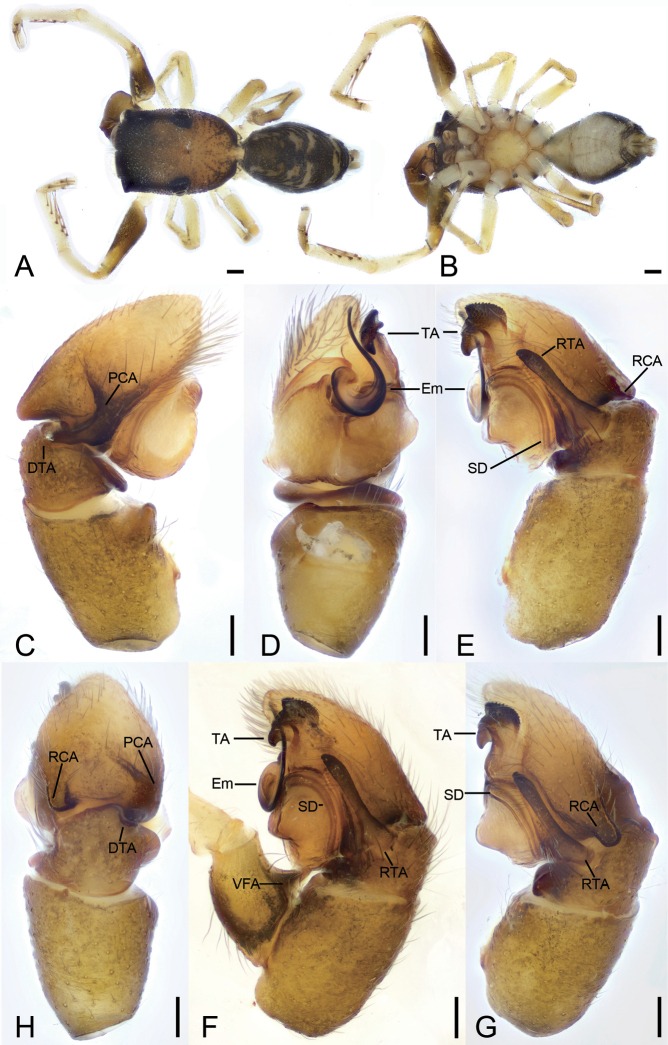
*Synagelidesjinding* sp. nov., holotype male **A** habitus, dorsal view **B** same, ventral view **C** palp, prolateral view **D** same, ventral view **E** same, retrolateral view **F** same, detail of ventral femoral apophysis, retrolateral view **G** same, retrolateral view, slightly dorsal **H** same, dorsal view. Abbreviations: DTA – dorso-prolateral tibial apophysis, Em – embolus, PCA – postero-prolateral cymbial apophysis, RCA – postero-retrolateral cymbial apophysis, RTA – retrolateral tibial apophysis, SD – sperm duct, SS – scale-like serrations, TA – terminal apophysis, VFA – ventral femoral apophysis. Scale bars: 0.2 mm (**A, B**); 0.05 mm (**C–E, G, H**); 0.1 mm (**F**).

**Figure 5.**
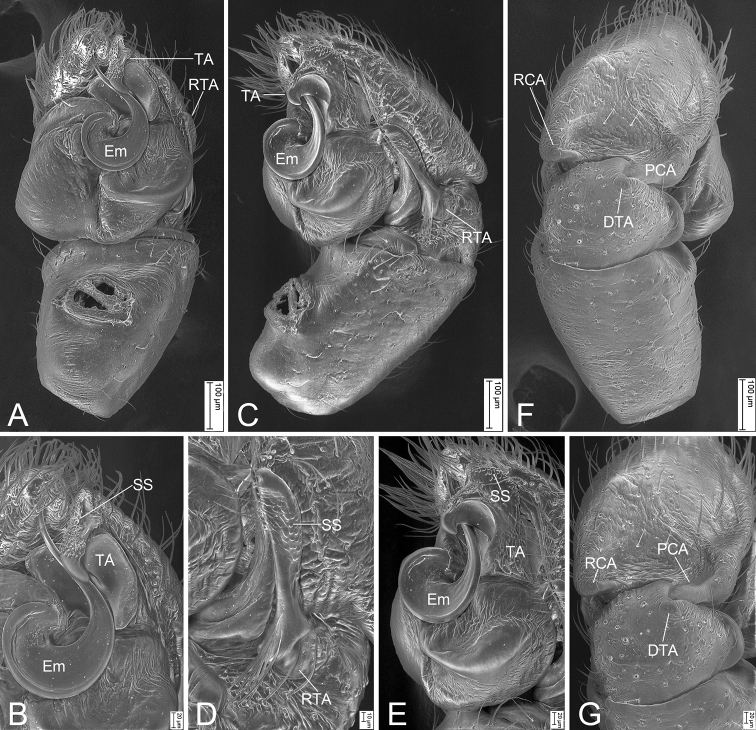
*Synagelidesjinding* sp. nov., SEMs of holotype male **A** palp, ventral view **B** same, detail of terminal apophysis and embolus, ventral view **C** same, retrolateral view **D** same, detail of retrolateral tibial apophysis, retrolateral view **E** same, detail of terminal apophysis and embolus, retrolateral view **F** same, dorsal view, slightly prolateral **G** same, detail of postero-prolateral cymbial apophysis, postero-retrolateral cymbial apophysis and dorso-prolateral tibial apophysis, dorsal view. Abbreviations: DTA – dorso-prolateral tibial apophysis, Em – embolus, PCA – postero-prolateral cymbial apophysis, RCA – postero-retrolateral cymbial apophysis, RTA – retrolateral tibial apophysis, SS – scale-like serrations, TA – terminal apophysis.

The male of this species is most similar to that of *Synagelidesannae* Bohdanowicz, 1979 (see Bohdanowicz 1979: 56, figs 14–17) in having a sharp ventral femoral apophysis, an anticlockwise spiral embolus, a C-shaped terminal apophysis with hook-shaped tip, and the mastoid tegular apophysis in retrolateral view, but differs from it in having (Figs 4C–H, 5) the posterior cymbium with a long blunt retrolateral apophysis (vs absent), the parallel retrolateral tibial apophysis together with postero-retrolateral cymbial apophysis in retrolateral view (vs. convergent) and thick clavate retrolateral tibial apophysis (vs spine-like) with many scale-like serrations (vs absent). It also resembles those seven species *S.birmanicus* Bohdanowicz, 1987 (see Bohdanowicz 1987: 84, figs 66–72), *S.cavaleriei* (Schenkel, 1963) (see Bohdanowicz 1987: 66, figs 1, 2), *S.gosainkundicus* Bohdanowicz, 1987 (see Bohdanowicz 1987: 78, figs 45, 46), *S.kosi* Logunov & Hereward, 2006 (see Logunov and Hereward 2006: 285, figs 21, 22), *S.martensi* Bohdanowicz, 1987 (see Logunov and Hereward 2006: 287, figs 37–40), *S.oleksiaki* Bohdanowicz, 1987 (see Bohdanowicz 1987: 79, figs 47, 48), and *S.walesai* Bohdanowicz, 1987 (see Bohdanowicz 1987: 72, figs 23, 24), but it can be easily distinguished from them by the parallel retrolateral tibial apophysis together with postero-retrolateral cymbial apophysis (vs convergent).

###### Description.

***Habitus*** as in Fig. 4A, B. Total length 2.97. Carapace 1.50 long, 1.09 wide. Eye sizes and interdistances: AME 0.30; ALE 0.18; PME 0.08; PLE 0.17; AME–AME 0.07; AME–ALE 0.04; PME–PME 0.77; ALE–ALE 0.73; PME–PLE 0.30; PLE–PLE 0.95; ALE–PLE 0.75; AME–PME 0.38; AME–PLE 0.63. MOA: 0.64 long; 0.67 anterior width, 0.91 posterior width. Fovea (Fig. 4A) round, hollowed. Chelicerae (Fig. 4B) with two promarginal teeth (proximal larger) and one large laminar retromarginal teeth. Sternum (Fig. 4B) shield-shaped, longer than wide, posterior end arch-shaped, smooth. Leg measurements: I 3.36 (1.07, 0.71, 0.9, 0.36, 0.32); II 2.2 (0.67, 0.32, 0.46, 0.47, 0.28); III 2.33 (0.69, 0.28, 0.5, 0.55, 0.31); IV 2.25 (0.65, 0.28, 0.52, 0.55, 0.25). Femur width: I 0.31; II 0.21; III 0.21; IV 0.18. Leg spination (Fig. 4A, B): I tipv 1-2-1, rv 1-2-1; Met pv 0-1-1, rv 0-1-1. Pedicel 0.09. Abdomen 1.37 long, 0.83 wide.

***Coloration*** (Fig. 4A, B). Carapace yellow-brown, anterior part darker than posterior, posteriorly with radial grooves and 12–14 rows of short black setae. Endites yellowish, mottled. Labium yellowish brown, anteriorly with a single row of strong setae, posteriorly mottled. Sternum, yellow, with pale brown mottled spots around margin. Legs: trochanters I–IV yellow, with dark brown stripe; femur I dark yellow-brown, femora II–IV yellow, with prolateral dark brown stripes; patellae, tibiae, and metatarsi yellow, with dark brown lateral stripes; tarsi yellowish, proximal part darker than distal. Abdomen yellowish to dark brown, with three pairs of yellowish stripes in anterior part and one arch-shaped, yellowish stripe on subposterior part; venter yellowish to yellow. Spinnerets yellowish brown, mottled.

***Palp*** (Figs 4C–H, 5). Femur with a strongly sharp, tooth-like ventral apophysis. Patella swollen, with a length–width ratio of ca 1.58. Tibia small and narrow, with a long strong clavate retrolateral apophysis which presents many little scale-like serrations on anterior surface and nearly longer than 1/2 length of cymbium, and a dorsal apophysis locking cymbial postero-prolateral apophysis. Cymbium bullet-shaped in dorsal view, with a long, strong, blunt, sclerotized postero-retrolateral and a long, strong, triangular, postero-prolateral apophysis. Tegulum broad, C-shaped extended in ventral view, with a clear mastoid apophysis and a thin sperm duct in retrolateral view. Terminal apophysis arising from antero-retrolateral part of tegulum, strongly sclerotized, Y-shaped in retrolateral view, with abundant little scale-like serrations on antero-retrolateral surface. Embolus with an anticlockwise spiral in ventral view, longer than terminal apophysis, with broad convoluted basal part and whip-shaped apical part.

**Female.** Unknown.

###### Comments.

The male of this species is not conspecific with the female of *Synagelidestriangulatus* sp. nov. for the following reasons. Firstly, the male abdomen has the two pairs of white stripes medially (vs a pair of spots and one chevron-shaped yellowish stripe in *S.triangulatus*) and the arch-shaped yellowish stripe located subposteriorly (vs absent in *S.triangulatus*).

###### Distribution.

Known only from the type locality in Jiangxi Province, China (Fig. 13).

##### ﻿ Synagelides serratus

Taxon classificationAnimaliaAraneaeSalticidae

Liu sp. nov.

23562314-3A37-51E7-B6BF-17B82AA24453

http://zoobank.org/C6C0FB47-5AF8-43D9-9C3B-86E5239E4BF8

Figs 6 , 7 , 8

###### Material examined.

***Holotype*** ♂, 26°40'48.69"N, 115°25'07.79"E, 1031 m, Dawu Mountain, near Xilin Village, dawu Monuntain, Longjiatang Village, Donggu Town, Qingyuan District, Ji’an City, Jiangxi Province, China, 25 October 2020, K. Liu, Y. Ying & S. Yuan leg. ***Paratype*** 1 ♀, the same data as holotype.

###### Etymology.

The name from the Latin word *serratus*, referring to the saw-like retrolateral apophysis; adjective.

###### Diagnosis.

**Figure 6.**
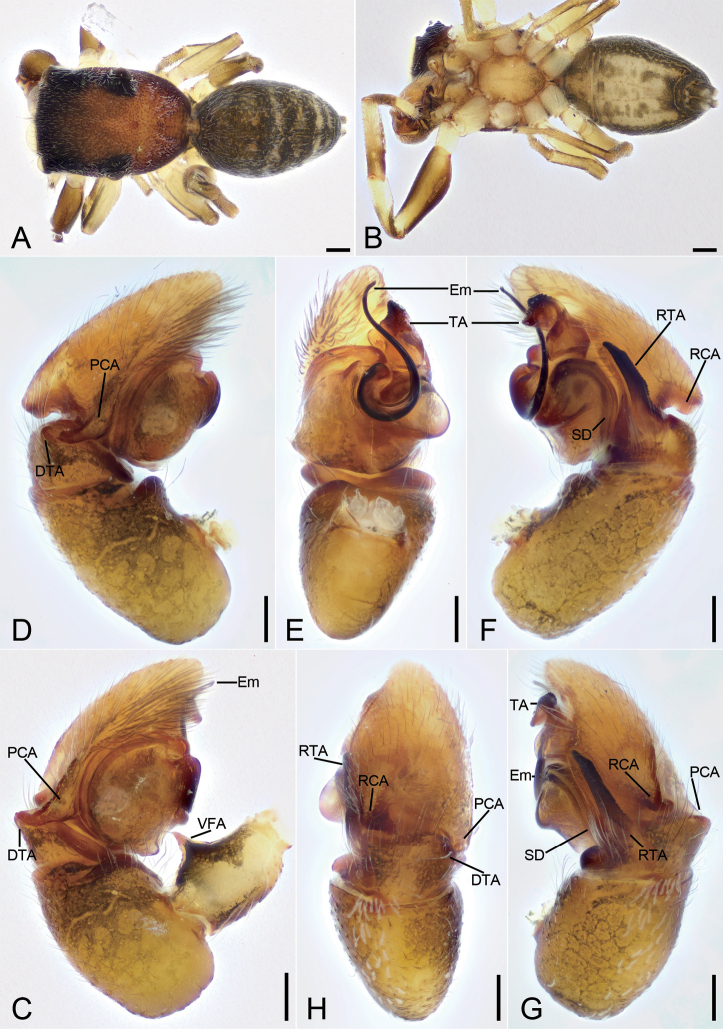
*Synagelidesserratus* sp. nov., holotype male **A** habitus, dorsal view **B** same, ventral view **C** palp, detail of ventral femoral apophysis, prolateral view **D** same, prolateral view **E** same, ventral view **F** same, retrolateral view **G** same, retrolateral view, slightly dorsal **H** same, dorsal view. Abbreviations: DTA – dorso-prolateral tibial apophysis, Em – embolus, PCA – postero-prolateral cymbial apophysis, RCA – postero-retrolateral cymbial apophysis, RTA – retrolateral tibial apophysis, SD – sperm duct, SS – scale-like serrations, TA – terminal apophysis, VFA – ventral femoral apophysis. Scale bars: 0.2 mm (**A, B**); 0.1 mm (**C**); 0.05 mm (**D–H**).

**Figure 7.**
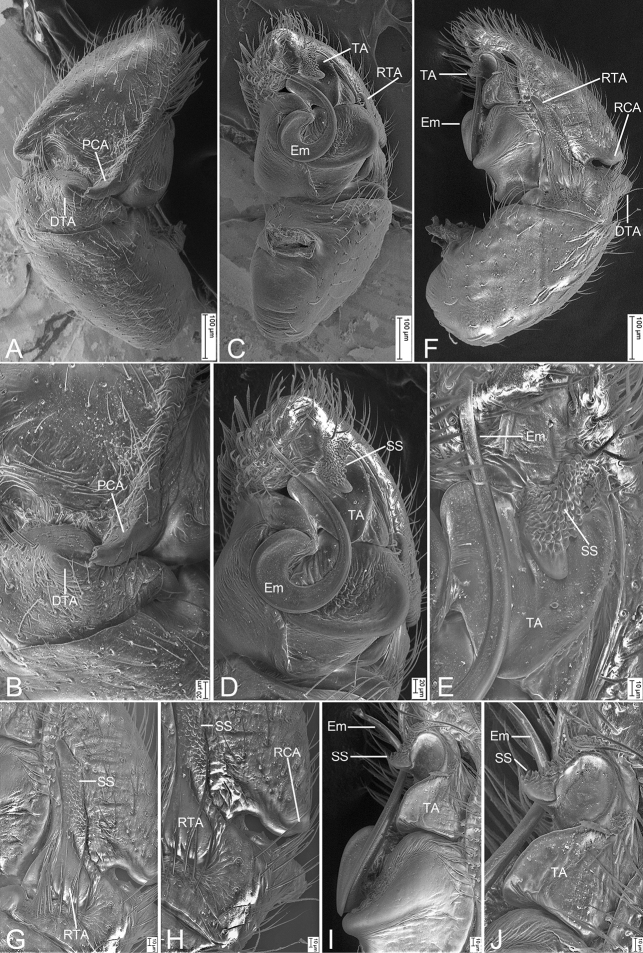
*Synagelidesserratus* sp. nov., SEMs of holotype male **A** palp, prolateral view, strongly dorsal **B** same, detail of postero-prolateral cymbial apophysis and dorso-prolateral tibial apophysis, prolateral view, strongly dorsal **C** same, retrolateral view, slightly retrolateral **D** same, detail of retrolateral tibial apophysis, ventral view, slightly retrolateral **E** same, detail of terminal apophysis and embolus, ventral view, slightly retrolateral **F** same, retrolateral view **G** same, detail of retrolateral tibial apophysis, retrolateral view **H** same, detail of retrolateral tibial apophysis and postero-retrolateral cymbial apophysis, retrolateral view **I** same, detail of terminal apophysis and embolus, retrolateral view **J** same, detail of terminal apophysis and embolus, retrolateral view. Abbreviations: DTA – dorso-prolateral tibial apophysis, Em – embolus, PCA – postero-prolateral cymbial apophysis, RCA – postero-retrolateral cymbial apophysis, RTA – retrolateral tibial apophysis, SS – scale-like serrations, TA – terminal apophysis.

**Figure 8.**
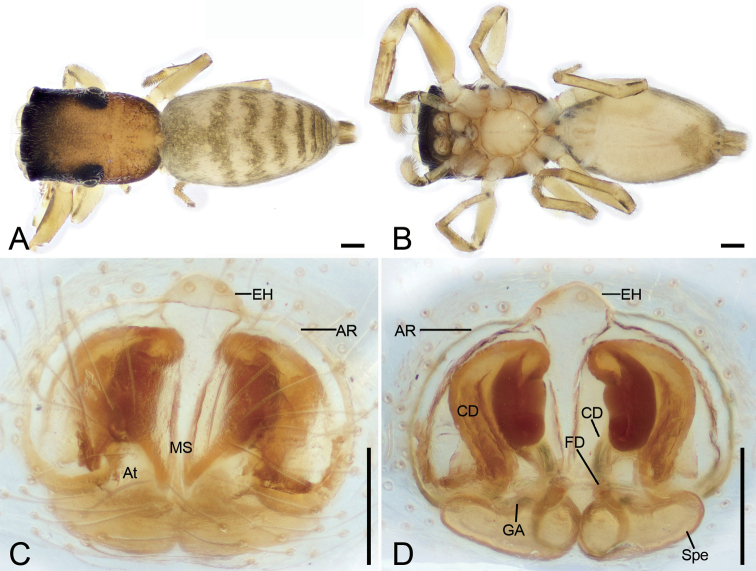
*Synagelidesserratus* sp. nov., female paratype **A** habitus, dorsal view **B** same, ventral view **C** epigyne, ventral view **D** same, dorsal view. Abbreviations: AR – atrial rim, At – atrium, CD – copulatory duct, CO – copulatory opening, EH – epigynal hood, FD – fertilization duct, GA – glandular appendages, MS – median septum, Spe – spermatheca. Scale bars: 0.2 mm (**A, B**); 0.05 mm (**C, D**).

The male of this species is similar to that of *Synagelidesannae* in having an anticlockwise spiral embolus and a C-shaped terminal apophysis (see Bohdanowicz 1979: 56, figs 14–17), but differs from it in having (Figs 6C–H, 7) the posterior cymbium with a short blunt retrolateral apophysis (vs absent), the femur with a spine-like ventral apophysis (vs relatively broadly triangular) in prolateral view, and the saw-like retrolateral tibial apophysis in retrolateral view (vs long and spine-like). It also resembles seven species, *S.birmanicus* Bohdanowicz, 1987 (see Bohdanowicz 1987: 84, figs 66–72), *S.cavaleriei* (Schenkel, 1963) (see Bohdanowicz 1987: 66, figs 1, 2), *S.gosainkundicus* Bohdanowicz, 1987 (see Bohdanowicz 1987: 78, figs 45, 46), *S.kosi* Logunov & Hereward, 2006 (see Logunov and Hereward 2006: 285, figs 21, 22), *S.martensi* Bohdanowicz, 1987 (see Logunov and Hereward 2006: 287, figs 37–40), *S.oleksiaki* Bohdanowicz, 1987 (see Bohdanowicz 1987: 79, figs 47, 48), and *S.walesai* Bohdanowicz, 1987 (see Bohdanowicz 1987: 72, figs 23, 24), but can be easily distinguished from them by the very short postero-retrolateral cymbial apophysis (vs relatively long). The female of this species resembles that of *S.cavaleriei* in the anteromedially located, bell-shaped epigynal hood and the elongated, touching spermathecae (see Peng 2020: 446, fig. 325a, b), but it can be easily separated in having (Fig. 8C, D) the copulatory openings located subposteromedially (vs medially) and the broad part of copulatory ducts extending like a question mark (vs double C-shaped mark).

###### Description.

**Male** (holotype, sp1-20201025-4, red label). ***Habitus*** as in Fig. 6A, B. Total length 2.89. Carapace 1.40 long, 0.97 wide. Eye sizes and interdistances: AME 0.26; ALE 0.15; PME 0.04; PLE 0.15; AME–AME 0.07; AME–ALE 0.05; PME–PME 0.77; ALE–ALE 0.69; PME–PLE 0.26; PLE–PLE 0.82; ALE–PLE 0.51; AME–PME 0.33; AME–PLE 0.45. MOA: 0.45 long; 0.58 anterior width, 0.85 posterior width. Fovea (Fig. 6A) round, hollowed. Chelicerae (Fig. 6B) with two promarginal teeth (proximal larger) and one large laminar retromarginal teeth. Sternum (Fig. 6B) shield-shaped, longer than wide, posterior end arch-shaped, smooth. Leg measurements: I 2.79 (0.87, 0.62, 0.7, 0.35, 0.25); II 1.93 (0.56, 0.33, 0.39, 0.38, 0.27); III 2.08 (0.64, 0.29, 0.4, 0.48, 0.27); IV 2.46 (0.72, 0.32, 0.52, 0.62, 0.28). Femur width: I 0.35; II 0.20; III 0.21; IV 0.18. Leg spination (Fig. 6A, B): I tipv 2-1-1, rv 2-1-1; Met pv 1-1-0, rv 1-1-0. Pedicel 0.03. Abdomen 1.47 long, 0.89 wide.

***Coloration*** (Fig. 6A, B). Carapace reddish brown, anterior part darker than posterior, posteriorly with radial grooves, and 10–14 rows of short scale-like white setae. Endites yellow, mottled. Labium yellow-brown, anteriorly with a single row of strong setae, posteriorly mottled. Sternum yellow with pale brown, mottled spots around margin. Legs: trochanters I–IV yellow, with dark brown stripe; femur I dark yellow-brown, femora II–IV yellow, with prolateral dark brown stripes; patellae, tibiae, and metatarsi yellow, with dark brown lateral stripes; tarsi yellowish. Abdomen dark brown, mottled, with four chevron-shaped yellowish stripes on posterior part; venter with many irregular dark brown spots. Spinnerets dark yellow-brown, mottled.

***Palp*** (Figs 6C–H, 7). Femur with a very sharp, spine-like, ventral apophysis. Patella swollen, with a length–width ratio of ca 1.92. Tibia small and narrow, with a long, strong, saw-like retrolateral apophysis which presents many scale-like serrations on lateral surface and nearly as long as 1/2 length of cymbium, and a stubby dorsal apophysis locking cymbial postero-prolateral apophysis. Cymbium bullet-shaped in dorsal view, with a short, strong, blunt, sclerotized postero-retrolateral and a long, strong, triangular postero-prolateral apophysis. Tegulum very broad, with a clear mastoid apophysis in ventral view and a thin sperm duct in retrolateral view. Terminal apophysis C-shaped in retrolateral view, strongly sclerotized and curved, arising from antero-retrolateral part of tegulum, with abundant, little, scale-like serrations on distal surface. Embolus with an anticlockwise spiral in ventral view, longer than terminal apophysis, with relatively broad curved basal part and whip-shaped apical part.

**Female** (paratype, sp1-20201025-4, yellow label). ***Habitus*** as in Fig. 8A, B. As in male, except as noted. Total length 2.85. Carapace 1.24 long, 0.84 wide. Eye sizes and interdistances: AME 0.23; ALE 0.13; PME 0.05; PLE 0.12; AME–AME 0.10; AME–ALE 0.08; PME–PME 0.72; ALE–ALE 0.62; PME–PLE 0.23; PLE–PLE 0.78; ALE–PLE 0.53; AME–PME 0.38; AME–PLE 0.66. MOA: 0.56 long; 0.54 anterior width, 0.81 posterior width. Sternum (Fig. 8B), posterior end triangular, relatively blunt. Leg measurements: I 2.16 (0.7, 0.45, 0.54, 0.26, 0.21); II 1.56 (0.51, 0.16, 0.35, 0.29, 0.25); III 1.84 (0.55, 0.28, 0.36, 0.38, 0.27); IV 2.45 (0.7, 0.31, 0.59, 0.58, 0.27). Femur width: I 0.31; II 0.20; III 0.21; IV 0.21. Pedicel 0.11. Leg spination (Fig. 8A, B): I tipv 2-2-0, rv 2-2-0; Met pv 1-0-1, rv 1-0-1. Pedicel 0.11. Abdomen 1.55 long, 0.92 wide.

***Coloration*** (Fig. 8A, B). Paler than male. Carapace yellow-brown. Sternum yellowish, posteromedially with mottled dark brown stripe. Abdomen yellowish, with two transverse brown stripes in anterior part, two chevron-shaped brown stripes medially, and four transverse brown stripes posteriorly; venter yellowish, with a V-shaped marking medially and a large brown spot posteriorly.

***Epigyne*** (Fig. 8C, D). Epigynal plate cap-shaped, with a short median septum. Epigynal hood broadly bell-shaped, arising from anteromedial atrial rim. Atrium small, widely separated. Atrial rim round, slightly sclerotized. Copulatory ducts very long, anterior part like a question mark, posterior part tubed with a slight curve medially, connecting with subposterior part of spermathecae. Glandular appendages very short, near the base of fertilization ducts. Spermathecae large, elongated, swollen, closely touching. Fertilization ducts relatively long, nearly as long as 1/2 length of spermathecae, transversely extended.

###### Distribution.

Known only from the type locality in Jiangxi Province, China (Fig. 13).

##### ﻿ Synagelides shuqiang

Taxon classificationAnimaliaAraneaeSalticidae

Liu sp. nov.

86D875BF-189C-5BF0-87EB-065AACCC502F

http://zoobank.org/1FC0A541-AB4C-40FB-85BC-B01AC3290902

Figs 9 , 10 , 11

###### Material examined.

***Holotype*** ♂, 24°55'35.36"N, 115°27'25.09"E, 716 m, Guiz­humao Parking lot, near the county-boundary between Xunwu and Anyuan County, Ganzhou City, Jiangxi Province, China, 7 October 2020, K. Liu, Y. Ying, M. Zhang & J. Yan leg.

###### Etymology.

The species is named in honor of Dr Shuqiang Li, a well-known arachnologist (Institute of Zoology, Chinese Academy of Sciences, Beijing); noun in apposition.

###### Diagnosis.

**Figure 9.**
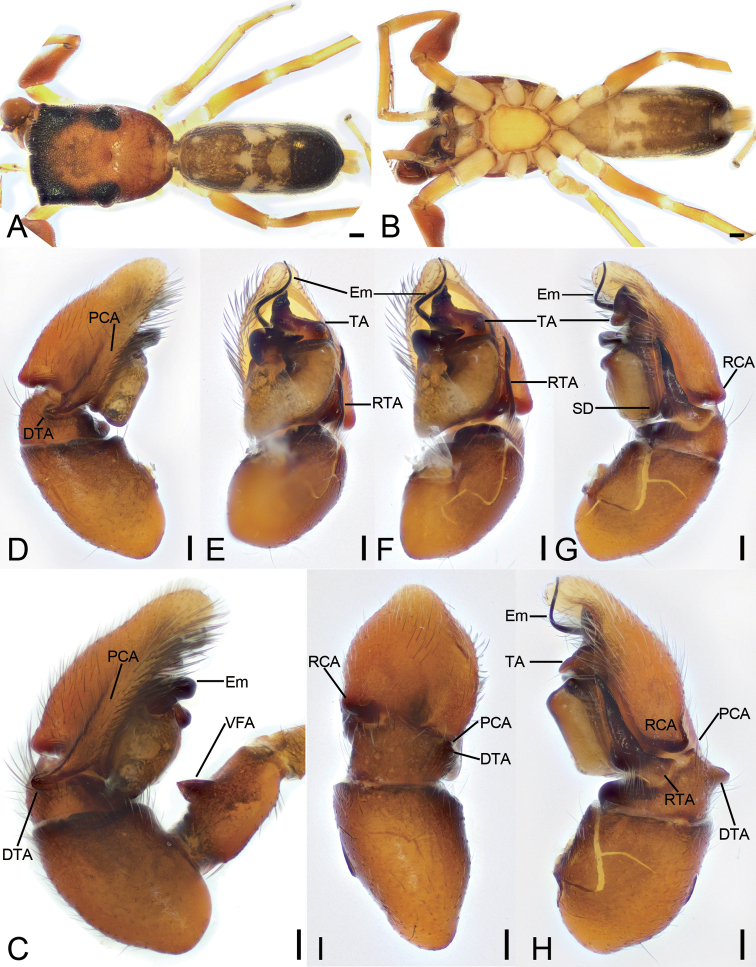
*Synagelidesshuqiang* sp. nov., holotype male **A** habitus, dorsal view **B** same, ventral view **C** palp, detail of ventral femoral apophysis, prolateral view **D** same, prolateral view, slightly dorsal **E** same, ventral view **F** same, ventral view, slightly retrolateral **G** same, retrolateral view **H** same, retrolateral view, slightly dorsal **I** same, dorsal view. Abbreviations: DTA – dorso-prolateral tibial apophysis, Em – embolus, PCA – postero-prolateral cymbial apophysis, RCA – postero-retrolateral cymbial apophysis, RTA – retrolateral tibial apophysis, SD – sperm duct, SS – scale-like serrations, TA – terminal apophysis, VFA – ventral femoral apophysis. Scale bars: 0.2 mm (**A, B**); 0.1 mm (**C**); 0.05 mm (**D–I**).

**Figure 10.**
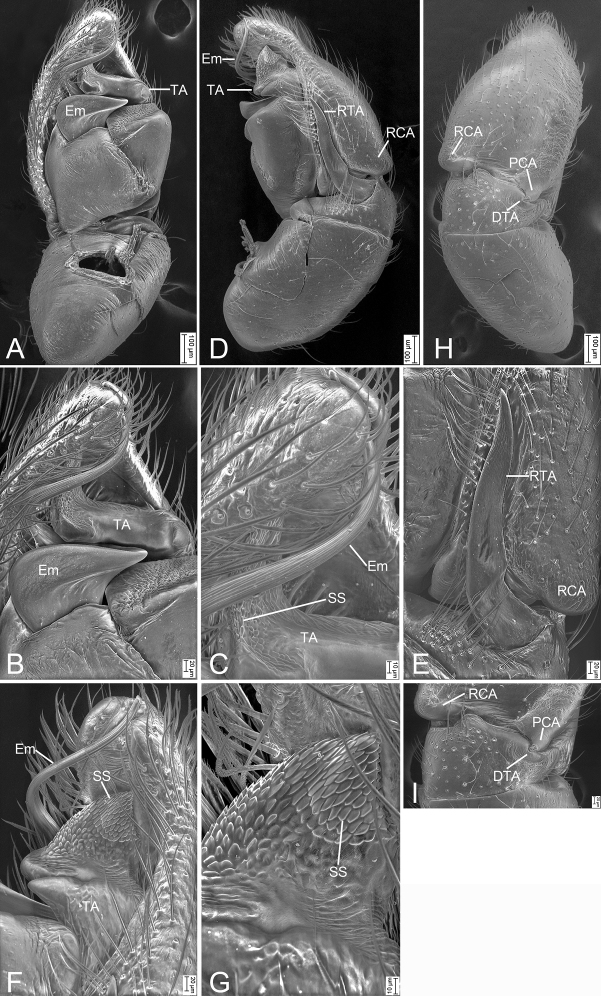
*Synagelidesshuqiang* sp. nov., SEMs of holotype male **A** palp, ventral view **B** same, detail of terminal apophysis and embolus, ventral view **C** same, detail of terminal apophysis and embolus, ventral view **D** same, retrolateral view **E** same, detail of retrolateral tibial apophysis, retrolateral view **F** same, detail of terminal apophysis and embolus, retrolateral view **G** same, detail of terminal apophysis, retrolateral view **H** same, dorsal view **I** same, detail of postero-prolateral cymbial apophysis, postero-retrolateral cymbial apophysis and dorso-prolateral tibial apophysis, dorsal view. Abbreviations: DTA – dorso-prolateral tibial apophysis, Em – embolus, PCA – postero-prolateral cymbial apophysis, RCA – postero-retrolateral cymbial apophysis, RTA – retrolateral tibial apophysis, SS – scale-like serrations, TA – terminal apophysis.

The male of this species is most similar to that of *Synagelideshamatus*Zhu et al. 2005 (Zhu et al. 2005: 541, fig. 12D, E) and *S.palpalis* Żabka, 1985 (Wang et al. 2020: 16, fig. 17D) in having a convoluted embolus reaching cymbial tip and the shape of tegulum, but differs from them in having (Figs 9C–I, 10) a L-shaped terminal apophysis in ventral view (vs broadly hook-shaped in *S.hamatus* and S-shaped in *S.palpalis*) and the sword-shaped retrolateral tibial apophysis (vs forked in *S.hamatus* and spine-like in *S.palpalis*) slightly longer than 1/2 length of cymbium (vs much longer than 1/2 length of cymbium in *S.hamatus* and *S.palpalis*) in retrolateral view. It also resembles seven species, *S.birmanicus* Bohdanowicz, 1987 (see Bohdanowicz 1987: 84, figs 66–72), *S.cavaleriei* (Schenkel, 1963) (see Bohdanowicz 1987: 66, figs 1, 2), *S.gosainkundicus* Bohdanowicz, 1987 (see Bohdanowicz 1987: 78, figs 45, 46), *S.kosi* Logunov & Hereward, 2006 (see Logunov and Hereward 2006: 285, figs 21, 22), *S.martensi* Bohdanowicz, 1987 (see Logunov and Hereward 2006: 287, figs 37–40), *S.oleksiaki* Bohdanowicz, 1987 (see Bohdanowicz 1987: 79, figs 47, 48), and *S.walesai* Bohdanowicz, 1987 (see Bohdanowicz 1987: 72, figs 23, 24), but can be easily distinguished from them by the very short and broad postero-retrolateral cymbial apophysis in retrolateral view (vs relatively long and thin) and the triangular terminal apophysis in retrolateral view (vs C-shaped).

###### Description.

**Figure 11.**
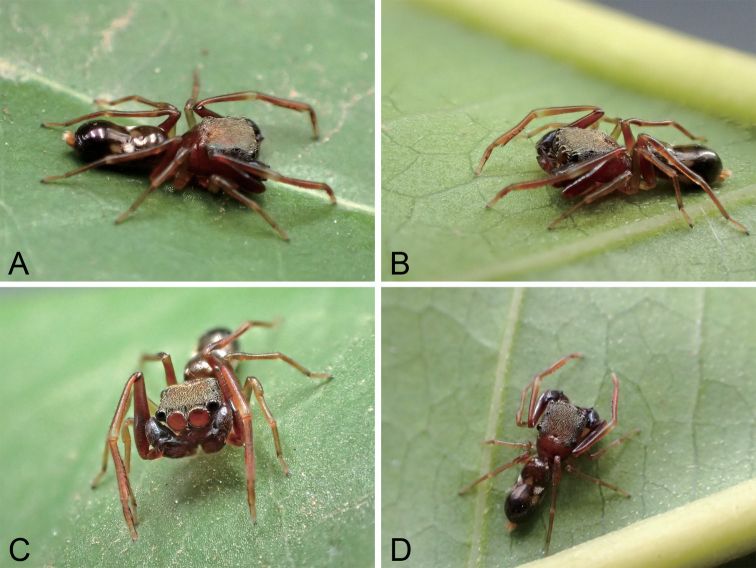
Photographs of living male specimens of *Synagelidesshuqiang* sp. nov., from Ganzhou City in Jiangxi Province, China.

***Habitus*** as in Figs 9A, B, 11. Total length 4.63. Carapace 1.98 long, 1.44 wide. Eye sizes and interdistances: AME 0.48; ALE 0.23; PME 0.13; PLE 0.25; AME–AME 0.10; AME–ALE 0.10; PME–PME 1.14; ALE–ALE 1.06; PME–PLE 0.42; PLE–PLE 1.24; ALE–PLE 0.83; AME–PME 0.48; AME–PLE 0.96. MOA: 0.74 long; 0.92 anterior width, 1.28 posterior width. Fovea (Fig. 9A) round, hollowed. Chelicerae (Fig. 9B) with two promarginal teeth (proximal larger) and one large laminar retromarginal teeth. Sternum (Fig. 9B) shield-shaped, longer than wide, anterolateral sloping, posterior end arch-shaped. Leg measurements: I 5.03 (1.54, 1.44, 1.24, 0.49, 0.32); II 3.37 (1.03, 0.51, 0.83, 0.69, 0.31); III 3.25 (0.97, 0.48, 0.78, 0.68, 0.34); IV 4.35 (1.25, 1.29, 0.57, 0.84, 0.4). Femur width: I 0.42; II 0.26; III 0.28; IV 0.28. Leg spination (Fig. 9A, B): I tipv 0-3-1, rv 0-3-1; Met pv 1-0-1, rv 1-0-1. Pedicel 0.20. Abdomen 2.46 long, 1.01 wide.

***Coloration*** (Fig. 9A, B). Carapace reddish brown, anterior part darker than posterior, posteriorly with radial grooves, and 12–16 rows of short scale-like, black setae. Endites yellow-brown, mottled. Labium yellow-brown, anteriorly with a single row of strong setae, posteriorly dark brown. Sternum, yellow, with pale brown mottled spots around margin. Legs: trochanter I yellow, trochanters II–IV yellowish; femur I reddish brown, femora II–IV yellow; tibiae, patellae, and metatarsi yellow; tarsi yellowish. Abdomen dark brown, mottled, with one broad yellowish stripe including a semicircular dark brown marking in medial part; venter yellow to dark brown, with three dark brown adjacent stripes, posterior part fusing. Spinnerets dark yellow.

***Palp*** (Figs 9C–I, 10). Femur with a thick, strong, tooth-like ventral apophysis. Patella swollen, with a length–width ratio of ca 1.76. Tibia small and narrow, with a long, strong, sword-like, retrolateral apophysis which slightly longer than 1/2 length of cymbium and a ridge-like prolateral apophysis locking cymbial postero-prolateral apophysis. Cymbium bullet-shaped in dorsal view, with a short, strong, broad, sclerotized postero-retrolateral and a long, strong, thick postero-prolateral apophysis. Tegulum very broad, lacking mastoid apophysis in ventral view, with a thin sperm duct in retrolateral view. Terminal apophysis strongly sclerotized, L-shaped, and with a horn-like tip in ventral view, arising from antero-retrolateral part of tegulum, with abundant strong, scale-like serrations on anterior surface. Embolus an anticlockwise convolute in ventral view, longer than terminal apophysis, with relatively broad curved basal part, and whip-shaped apical part, apex extending beyond the cymbial tip.

**Female.** Unknown.

###### Comments.

**Figure 12.**
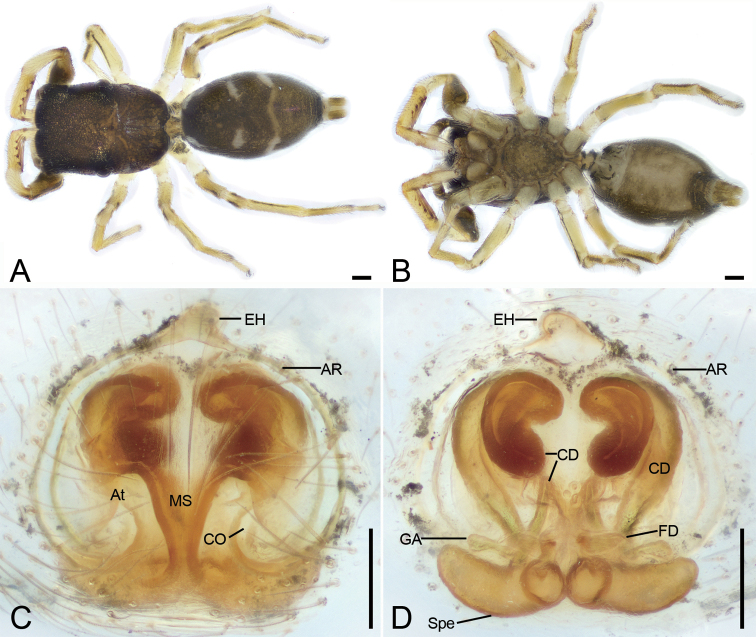
*Synagelidestriangulatus* sp. nov., holotype female **A** habitus, dorsal view **B** same, ventral view **C** epigyne, ventral view **D** same, dorsal view. Abbreviations: AR – atrial rim, At – atrium, CD – copulatory duct, CO – copulatory opening, EH – epigynal hood, FD – fertilization duct, GA – glandular appendages, MS – median septum, Spe – spermatheca. Scale bars: 0.2 mm (**A, B**); 0.05 mm (**C, D**).

The male of this species is not conspecific with *Synagelidestriangulatus* sp. nov. based on the following observations. Firstly, the male abdomen is elongated in dorsal view, nearly 2.5 times as long as wide, while in *S.triangulatus*, the length–width ratio is ca 1.5. Secondly, the abdomen has a clear constriction located medially (Fig. 11), but in the latter a constriction is absent (Fig. 12A, B). This species seems more successful than the latter in ant mimicry based on its habitus.

###### Distribution.

**Figure 13.**
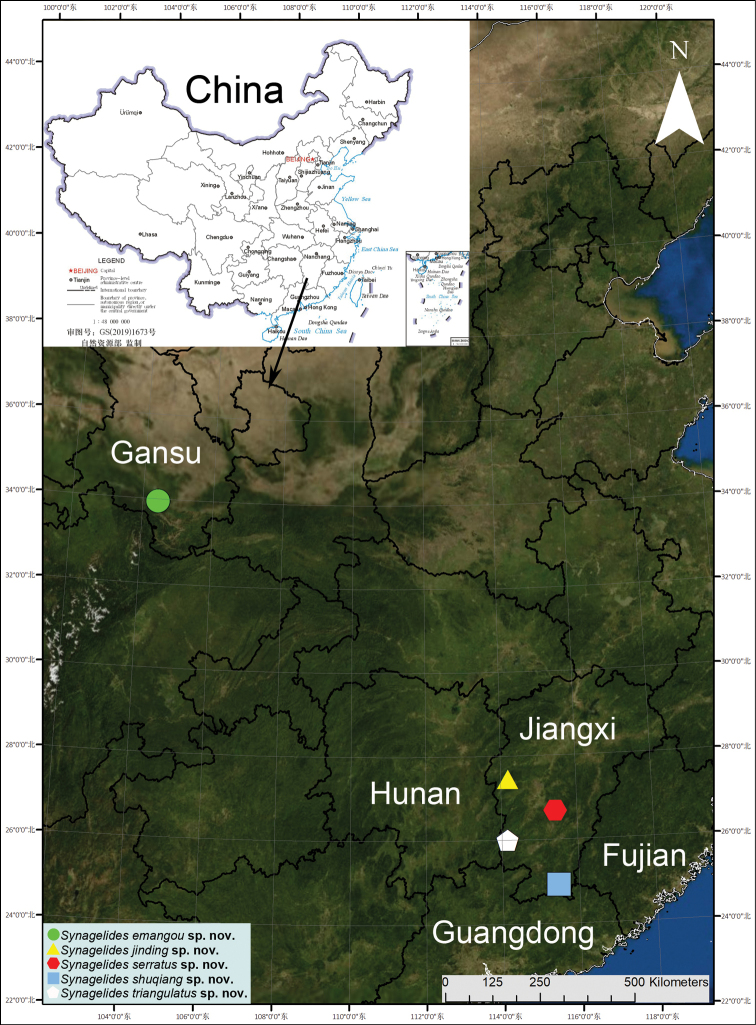
Records of *Synagelidesemangou* sp. nov. from Gansu province; *S.jinding* sp. nov., *S.serratus* sp. nov., *S.shuqiang* sp. nov., and *S.triangulatus* sp. nov. from Jiangxi Province in China.

Known only from the type locality in Jiangxi Province, China (Fig. 13).

##### ﻿ Synagelides triangulatus

Taxon classificationAnimaliaAraneaeSalticidae

Liu sp. nov.

B50EF395-0D48-5153-830B-C7A2F4C51A9C

http://zoobank.org/25E61438-A86C-4156-AFC9-569E31D87ED0

Fig. 12

###### Material examined.

***Holotype*** ♀, 26°00'28.25"N, 114°08'47.43"E, 1046 m, near Viewing Platform, Wuzhifeng Scenic Spot, Wuzhifeng Town, Shangyou County, Ganzhou City, Jiangxi Province, China, 1 October 2020, K. Liu, Y. Ying, M. Zhang & J. Yan leg. ***Paratype*** 1 subadult male, the same data as holotype.

###### Etymology.

The name is from the Latin word *triangulatus*, referring to the shape of the median septum; adjective.

###### Diagnosis.

The female of this species is most similar to *Synagelideskosi* Logunov & Hereward, 2006 (Logunov and Hereward 2006: 285, figs 24, 32) and *S.jinggangshanensis*Liu et al., 2017 (Liu et al. 2017: 292, figs 1C, D, 2A, B; holotype examined) in having the C-shaped median part and the sloping, slender, tub-shaped posterior part of copulatory duct, but can be separated from them by (Fig. 12C, D) the broad, bell-shaped epigynal hood (vs relatively thin in *S.kosi* and *S.jinggangshanensis*), the relatively broad triangular median septum (vs nearly T-shaped in *S.kosi* and and *S.jinggangshanensis*), and the closely touching spermathecae (vs slightly separated in *S.kosi* and *S.jinggangshanensis*). It also resembles seven other species, *S.annae* (see Bohdanowicz 1979: 56, figs 14–17), *S.birmanicus* Bohdanowicz, 1987 (see Bohdanowicz 1987: 84, figs 66–72), *S.cavaleriei* (Schenkel, 1963) (see Bohdanowicz 1987: 66, figs 1, 2), *S.gosainkundicus* Bohdanowicz, 1987 (see Bohdanowicz 1987: 78, figs 45, 46), *S.martensi* Bohdanowicz, 1987 (see Logunov and Hereward 2006: 287, figs 37–40), *S.oleksiaki* Bohdanowicz, 1987 (see Bohdanowicz 1987: 79, figs 47, 48), and *S.walesai* Bohdanowicz, 1987 (see Bohdanowicz 1987: 72, figs 23, 24), but can be easily distinguished from them in having the spermathecae as long as median septum (vs shorter or longer).

###### Description.

***Habitus*** as in Fig. 12A, B. Total length 3.16. Carapace 1.39 long, 1.01 wide. Eye sizes and interdistances: AME 0.23; ALE 0.16; PME 0.06; PLE 0.16; AME–AME 0.09; AME–ALE 0.08; PME–PME 0.81; ALE–ALE 0.76; PME–PLE 0.26; PLE–PLE 0.89; ALE–PLE 0.70; AME–PME 0.37; AME–PLE 0.57. MOA: 0.55 long; 0.64 anterior width, 0.90 posterior width. Fovea (Fig. 12A) round, hollowed. Chelicerae (Fig. 12B) with two promarginal teeth (proximal larger) and one large laminar retromarginal teeth. Sternum (Fig. 12A, B) shield-shaped, longer than wide, posterior end arch-shaped. Leg measurements: I 2.97 (0.93, 0.68, 0.75, 0.33, 0.28); II 1.88 (0.6, 0.28, 0.3, 0.4, 0.3); III 2.34 (0.67, 0.37, 0.46, 0.57, 0.27); IV 3.06 (0.79, 0.39, 0.81, 0.73, 0.34). Femur width: I 0.29; II 0.17; III 0.18; IV 0.25. Leg spination (Fig. 12A, B): I tipv 2-2-1, rv 2-2-0; Met pv 1-0-1, rv 1-0-1. Pedicel 0.18. Abdomen 1.57 long, 0.96 wide.

***Coloration*** (Fig. 12A, B). Carapace dark reddish brown, anterior part darker than posterior, posteriorly with radial grooves, 14–16 rows of short black setae. Endites yellow-brown, mottled. Labium yellow-brown, anteriorly with two rows of strong setae. Sternum, yellow-brown, mottled, with dark brown, mottled stripes around margin. Legs: trochanters yellow, with dark brown stripe; femur I dark yellow-brown, femora II–IV yellow, with distinct prolateral and retrolateral dark brown stripes; patellae, tibiae, and metatarsi yellow, with dark brown lateral stripes; tarsi yellow. Abdomen dark yellow-brown, mottled, with three clear white spots consisting of abundant white setae antero-laterally and three chevron-shaped stripes (the medial one clear, others indistinct) medially; venter with many irregular yellow-brown spots postero-medially. Spinnerets yellow-brown, mottled.

***Epigyne*** (Fig. 12C, D). Epigynal plate apple-shaped, with a triangular median septum. Epigynal hood broadly bell-shaped, arising from anteromedial atrial rim. Atrium relatively large, separated by the median septum. Atrial rim round, slightly sclerotized. Copulatory ducts very long, anterior part like a question mark, medial part C-shaped, posterior part slender tube-shaped, connecting with subposterior part of spermathecae. Glandular appendages long, near the base of fertilization ducts, shorter than 1/2 length of spermathecae. Spermathecae large, elongated, swollen, closely touching, posteriorly globular. Fertilization ducts relatively broad, nearly as long as 1/3 length of spermathecae, transversely extended.

**Male.** Unknown.

###### Distribution.

Known only from the type locality in Jiangxi Province, China (Fig. 13).

## Supplementary Material

XML Treatment for Synagelides emangou

XML Treatment for Synagelides jinding

XML Treatment for Synagelides serratus

XML Treatment for Synagelides shuqiang

XML Treatment for Synagelides triangulatus
